# Visual–Semantic Processing and Reading Comprehension in Chinese Deaf and Hard-of-Hearing Students: Roles of Updating, Semantic Radical Awareness, and Daily Sign Language Use

**DOI:** 10.3390/bs16101910

**Published:** 2026-10-15

**Authors:** Yanfei Du, Zheng Wang, Fang Gu, Xiaohong Bao, Junshan He, Dan Yang

**Affiliations:** School of Special Education, Changchun University, Changchun 130022, China; 240805349@mails.ccu.edu.cn (Z.W.); guf@ccu.edu.cn (F.G.); baoxh@ccu.edu.cn (X.B.); 240805350@mails.ccu.edu.cn (J.H.); 220802310@mails.ccu.edu.cn (D.Y.)

**Keywords:** deaf and hard-of-hearing students, reading comprehension, visual–semantic processing, semantic radical awareness, updating, daily sign language use

## Abstract

Reading comprehension among deaf and hard-of-hearing (DHH) students may depend partly on how semantic information embedded in Chinese characters is used. This cross-sectional study examined associations among working-memory updating, semantic and phonetic radical awareness, and reading comprehension, with exploratory comparisons across broad communication-mode groups. Fifty-two DHH middle school students completed a 2-back task, radical-awareness measures, along with grade-specific reading tests. Reading scores were standardized within grade; balanced 2-back accuracy served as the updating index. Parallel and conditional indirect-association models were estimated with 5000 bootstrap resamples. Updating was positively associated with both types of radical awareness but showed no significant total or direct association with reading comprehension. Semantic radical awareness was positively associated with reading comprehension and showed a significant specific statistical indirect association between updating and reading comprehension. The specific indirect association via phonetic radical awareness was not significant, whereas the contrast between the two specific indirect associations was significant. Exploratory moderation analyses indicated stronger associations among students who reported daily sign language use. Overall, these findings provide preliminary evidence of associations between visual–semantic processing, updating, communication mode, and reading comprehension among DHH students, and may inform instruction aimed at supporting their reading comprehension.

## 1. Introduction

Reading is essential to learning and social participation for deaf and hard-of-hearing (DHH) students. Their reading development is also associated with later emotional and behavioral adjustment (Stevenson et al., 2018). However, many DHH students experience substantial difficulty developing reading comprehension skills. Limited auditory input may weaken phonological representations and orthography–phonology mappings, thereby constraining reading development. Yet some skilled DHH readers achieve high levels of reading proficiency despite relatively weak phonological processing. A recent review of text reading comprehension in prelingually deaf readers found that lexical and syntactic skills, along with language ability across modalities, were associated with reading comprehension, whereas findings on phonological processing were mixed (Olujić Tomazin et al., 2025). Understanding reading development among DHH students, therefore, requires more than identifying the difficulties associated with limited auditory input. It also requires examining which forms of linguistic information are associated with stronger reading comprehension.

### 1.1. Visual–Semantic Processing and Reading Comprehension in DHH Readers

If weak phonological processing reflected a broader impairment across the reading system, these readers should also have difficulty recognizing words and accessing their meanings. Yet some DHH individuals maintain effective reading despite limited auditory input. This pattern suggests that they may draw on other sources of linguistic information and that their reading processes may be organized in distinctive ways. Research indicates that skilled deaf readers may assign different relative weights to orthographic, phonological, and semantic information than hearing readers. They can use orthographic and semantic information effectively while relying less on phonological coding (Bélanger et al., 2012; Emmorey & Lee, 2021; Sehyr & Emmorey, 2022). Neuroimaging evidence provides system-level support for this account. Skilled deaf readers and hearing readers matched on reading level engage broadly similar semantic networks during word-meaning processing, whereas the clearest group differences occur during phonological processing (Emmorey et al., 2013). This finding suggests that relatively weak phonological processing can coexist with effective semantic processing. The reading system of DHH readers may therefore not be globally weaker but may differ in its functional organization and relative weighting of orthographic, phonological, and semantic information.

Word-recognition studies focusing on orthographic processing reveal a similar distinction. Fariña et al. (2017) found that skilled deaf readers showed orthographic sensitivity comparable to that of hearing readers but did not exhibit the same phonological mediation effect. Eye-tracking research likewise showed that deaf readers obtained orthographic preview benefits during reading, whereas phonological preview benefits were observed mainly among skilled hearing readers (Bélanger et al., 2013). The same distinction is evident in reading comprehension. Among deaf and hearing adults matched on reading ability, Sehyr and Emmorey (2022) found that orthographic precision and semantic knowledge predicted reading comprehension among deaf readers, whereas phonological awareness did not.

The Triangle Model offers an account of these differences in the relative weighting of linguistic information. According to the model, reading does not rely on a single pathway but emerges from interactions among orthographic, phonological, and semantic representations. Word meaning can be accessed through joint mappings among orthography, phonology, and semantics or through a more direct connection between orthography and semantics. The relative contribution of each source of information may vary with learning experience and the statistical properties of the writing system (Harm & Seidenberg, 2004; Seidenberg & McClelland, 1989). The Lexical Quality Hypothesis complements this account by focusing on representational quality. It proposes that precise and stable lexical representations, together with high-quality connections among orthography, phonology, and semantics, support efficient meaning retrieval and reading comprehension (Perfetti, 2007; Perfetti & Hart, 2002). Together, these theories suggest that limited access to auditory–phonological information does not necessarily preclude effective access to meaning. If orthographic representations and orthography–semantics mappings are sufficiently precise and stable, DHH readers may still rely on them to access meaning. Under these conditions, visual word forms and their semantic connections may assume a greater role in reading.

Chinese phonetic compound characters provide a way to examine this possibility. In these characters, the semantic radical typically provides a cue to the meaning of the whole character, whereas the phonetic radical primarily provides information about pronunciation (L. Li et al., 2021; Y. Li et al., 2022). Both are components of Chinese orthographic structure, but they serve different linguistic functions. If DHH readers rely more heavily on connections between visual form and meaning when auditory–phonological information is limited, semantic radical awareness should be more closely associated with their reading comprehension. Research with Chinese deaf children has found that visual–orthographic processing is associated with reading performance and may serve as a link between general visual processing and reading ability (Zhao & Wu, 2021). A more recent study found that Chinese DHH children retained some visual–orthographic processing ability and suggested that visual processing and some orthographic measures were associated with reading performance (S. Li et al., 2026). These findings support the relevance of visual processing to reading among DHH children but do not distinguish the roles of semantic and phonological information within orthographic structure. Accordingly, the present study uses semantic radical awareness as an important indicator of visual–semantic information use and includes phonetic radical awareness for comparison, examining whether these two types of orthographic information show different patterns of association with reading comprehension.

### 1.2. Updating, Visual–Semantic Processing, and Reading Comprehension

In the broader reading literature, updating has been linked to individual differences in reading comprehension (Carretti et al., 2005). Recent research has further examined the relationship between specific updating components and reading comprehension (Linares & Pelegrina, 2023). These findings suggest that updating may contribute to reading comprehension through component-specific cognitive processes. How updating relates to the specific written-language processing that DHH readers rely on, however, has not been directly examined. Zhao and Wu (2021) found that the association between general visual processing and reading ability in Chinese DHH children was partly mediated by visual–orthographic processing, suggesting that domain-general processing resources may be linked to reading performance through abilities specific to the writing system. De Vita et al. (2021) likewise found that children who differed in updating performance also showed differences on multiple writing and spelling measures. Although these studies differ in their participants and tasks, together they suggest that the relationship between performance on general cognitive tasks and literacy outcomes needs to be explained with reference to specific processes of written-language processing. Accordingly, the present study explores whether the association between updating, as a domain-general processing resource, and reading comprehension requires the involvement of specific written-language processes.

### 1.3. Communication Mode as a Contextual Boundary Condition for Visual–Semantic Processing

DHH students differ in their communication mode: some primarily use spoken language, some use sign language, and others use both sign and spoken language. Different communication modes may be associated with language exposure, visual-communication experience, and opportunities for semantic processing. Sign languages convey linguistic information through the visual–spatial modality. Signers derive linguistic meaning from features such as hand shape, location, movement, and spatial relations. Previous research has linked sign language proficiency to reading performance among DHH readers. Andrew et al. (2014) found that sign language ability was associated with written sentence comprehension among deaf children and adolescents. Sehyr and Emmorey (2022) similarly found that American Sign Language comprehension and fingerspelling skills were associated with reading comprehension among deaf readers. At the same time, skilled deaf readers can use orthographic and semantic information effectively without relying heavily on fine-grained phonological coding (Bélanger et al., 2012; Emmorey & Lee, 2021). These findings raise the possibility that experience in deriving linguistic meaning through visual forms in sign language may shape how linguistic information is used when reading print. However, previous studies have focused primarily on the association between sign language proficiency and overall reading performance. Less is known about whether daily sign language use is associated with specific forms of visual–semantic processing.

In Chinese reading, semantic radicals provide a visual–semantic cue that can be examined directly. Although sign language and semantic radicals differ in their linguistic structure, both involve deriving linguistic meaning from visual form. This shared feature does not imply that the two are structurally equivalent. Instead, it raises the question of whether the association between semantic radical awareness and reading comprehension varies with daily sign language use. Direct evidence on this question is currently lacking among Chinese DHH students.

The present study, therefore, treats daily sign language use as a boundary condition and examines whether the association between semantic radical awareness and reading comprehension varies across different patterns of daily language use. We do not assume that sign language use directly produces a particular reading mechanism. Rather, we examine whether visual–semantic processing, as indexed by semantic radical awareness, is associated with reading comprehension in different ways across language-use backgrounds.

### 1.4. The Current Study

Building on the preceding theoretical and empirical considerations, the present study examined the associations among visual–semantic processing, updating, and reading comprehension among DHH students, as well as the role of daily sign language use in these associations. Specifically, the study addressed five exploratory research questions:

RQ1: Is semantic radical awareness associated with reading comprehension among DHH students?

RQ2: Is updating directly associated with reading comprehension among DHH students?

RQ3: Is updating associated with semantic and phonetic radical awareness among DHH students?

RQ4: Are there statistical indirect associations between updating and reading comprehension via semantic and phonetic radical awareness, and do these associations differ?

RQ5: Do the associations involving semantic radical awareness and reading comprehension differ between students who do and do not use sign language in daily life?

## 2. Materials and Methods

### 2.1. Participants

Participants were recruited using convenience sampling between June 2024 and August 2025. The initial sample comprised 55 DHH middle school students from three special education schools serving DHH students in Jilin Province, China. No standardized screening procedure was employed in the present study. Participants were selected on the basis of school records and teacher reports, and had the following characteristics: (1) a documented diagnosis of hearing loss from a qualified institution; (2) no intellectual disability or other developmental disorder that could interfere with task completion; and (3) normal or corrected-to-normal vision and the ability to understand and complete the study tasks. After excluding students who did not complete all study tasks or whose data were incomplete and therefore could not be included in the analyses, the final analytic sample consisted of 52 students aged 13–19 years (*M* = 16.08, *SD* = 1.61). Hearing loss severity was determined from existing classifications in school records and available audiological documentation. Participants were not reclassified for the present study. Demographic and hearing-related characteristics of the participants are presented in Table 1.

### 2.2. Measures

A 2-back task, a test of semantic radical awareness and phonetic radical awareness based on Chinese phonetic compound characters, and a Chinese reading comprehension test were used to assess updating, semantic and phonetic radical awareness, and reading comprehension, respectively.

#### 2.2.1. Updating Task

Updating was assessed using a letter 2-back task. Participants continuously monitored each stimulus and compared it with the stimulus presented two positions earlier. The task assessed the maintenance and updating of information in working memory.

The stimuli consisted of uppercase English letters. Each stimulus was displayed for 500 ms, followed by an interstimulus interval (ISI) of 1000 ms. Participants pressed the “F” key when the current letter matched the letter presented two positions earlier and the “J” key when it did not. The first two stimuli in each sequence served only to establish the comparison baseline; no response was required for these stimuli, and they were excluded from the analysis.

Participants first completed 18 practice trials (5 matching, 13 non-matching) under the same stimulus timing (500 ms per stimulus, 1000 ms inter-stimulus interval) and response rules as the 90-trial main task (30 matching, 60 non-matching). Task instructions combined oral explanation, written instructions, and sign-language support, followed by a demonstration by the experimenter. Advancement to the main task required at least 60% accuracy in practice. Participants who misunderstood the task or fell short of this criterion received repeated instructions and/or further practice, up to five times. After five attempts, those still below criterion were advanced at the experimenter’s discretion—contingent on being able to correctly restate the task rules and on responding in the correct direction throughout practice—with the decision recorded; otherwise the session was terminated. The test phase contained 90 scorable trials, excluding the two initial lead-in stimuli. These comprised 30 match trials (33.3%) and 60 nonmatch trials (66.7%), presented in a predetermined pseudorandom order. Incorrect responses and omissions were scored as errors. Because the two trial types differed in number, this study used balanced accuracy—the mean of the hit rate and the correct rejection rate—as the primary 2-back metric, while also reporting overall accuracy, hit rate, correct rejection rate, and miss rate. Overall accuracy was treated as an index sensitive to the scoring method. The Spearman–Brown–corrected split-half reliability of balanced accuracy across odd and even trials was 0.884. Although balanced accuracy showed good split-half reliability, its construct validity remains unverified because the study used only one 2-back task and no independent updating measure. We therefore interpret it as a task-performance index sensitive to updating demands. Its associations with semantic and phonetic radical awareness provide preliminary nomological support, but not direct validation.

#### 2.2.2. Orthographic Awareness Test for Chinese Phonetic Compound Characters

The test was developed for the present study based on paradigms used in previous research on Chinese orthographic awareness (He, 2013; Shu et al., 2000) and was adapted to the literacy level of DHH middle school students. It comprised two subtests assessing semantic radical awareness and phonetic radical awareness. Before formal administration, experts in special education and Chinese language and orthography reviewed the contents of the test. The review focused on the phonetic-compound structure of the target characters, the semantic relationships between semantic radicals and whole-character meanings, the phonological relationships between phonetic radicals and whole-character pronunciations, item wording, and response options. Several items were revised based on expert feedback before the final version of the test was established.

Semantic radical awareness: This subtest assessed participants’ ability to identify target Chinese characters using semantic information provided by their semantic radicals. Target characters were selected primarily from Chinese-language textbooks for Grades 7–9 published by the People’s Education Press. Their phonetic-compound structure and relationships between semantic radicals and whole-character meanings were verified against the Dictionary of Phonetic Compound Characters in Modern Chinese (Ni, 1982) and A Dictionary of Chinese Character Information (Shanghai Jiao Tong University Chinese Character Coding Group & Shanghai Chinese Pinyin Writing Research Group, 1988). Each item presented a two-character word in which the target character was replaced by its pinyin transcription and was accompanied by four candidate characters. The candidates had identical or similar pronunciations and, whenever possible, shared the same phonetic radical while differing in their semantic radicals. This design minimized the usefulness of phonological and phonetic-radical information, thereby encouraging the participants to rely primarily on semantic cues provided by the semantic radicals. The subtest contained 60 items. Each correct response received 1 point, resulting in a total score ranging from 0 to 60. Higher scores indicated greater semantic radical awareness. Cronbach’s α for this subtest was 0.871. For example, for the Chinese character “偷 miáo”, participants selected the correct character from the four alternatives: “瞄”, “喵”, “描”, and “媌”.

Phonetic radical awareness: This subtest assessed participants’ ability to use phonetic-radical information to infer the pronunciation of phonetic compound characters. Target characters were selected primarily from middle school Chinese-language textbooks and varied in the degree to which their phonetic radicals provided cues to the pronunciation of the whole character. Participants were asked to write down the pronunciation of each target character. If they were unable to provide the pinyin transcription, they could instead write a familiar homophonic character. This option minimized the influence of pinyin writing ability on test performance; however, a response was scored as correct only when the reported pronunciation matched the target pronunciation exactly. The subtest contained 60 items, with 1 point awarded for each correct response. Total scores ranged from 0 to 60, with higher scores indicating greater awareness of phonetic radicals. Cronbach’s α for this subtest was 0.937. For example, participants used the phonetic radical in Chinese character “锣” as a cue to identify or infer its pronunciation.

#### 2.2.3. Chinese Reading Comprehension Test

A researcher-developed Chinese reading comprehension test was used to assess students’ reading comprehension. A separate version was developed for each grade to account for differences in reading ability. Each version contained eight items and had a maximum score of 24. The versions were broadly comparable in terms of text genre, passage length, number of items, item structure, and scoring method. Students completed the version corresponding to their grade, with higher scores indicating better reading comprehension. Cronbach’s α for the test was 0.893.

The researchers developed a standardized scoring rubric based on the reference answers. The rubric specified the key scoring elements for each item and the criteria for different response levels. Incorrect, irrelevant, or missing responses received 0 points. Partially correct responses received partial credit based on the scoring elements included and the completeness of the response, whereas complete and accurate responses received full credit. Two raters independently scored all reading comprehension responses. Interrater reliability was assessed using a two-way mixed-effects model with absolute agreement. The intraclass correlation coefficient for the total reading comprehension score was 0.982, 95% CI [0.969, 0.990]. To ensure that the test content and items were appropriate, eight experts in special education participated in two rounds of Delphi evaluation. The reading materials and items were revised in accordance with their feedback.

### 2.3. Administration Procedures

Test administrators completed standardized training before data collection. The 2-back task, orthographic awareness, and reading comprehension assessments were then administered in a fixed order in quiet classrooms at the participating schools. All participants received standardized instructions. All task instructions were presented in writing and supplemented with spoken or signed explanations according to each student’s communication needs. Formal testing began only after the test administrator confirmed that each participant fully understood the task rules. The complete session lasted approximately 35–45 min. Permission to conduct the study was obtained from all the participating schools.

Before testing, the researchers explained the purpose of the study, the assessment content and procedures, the voluntary nature of participation, the right to withdraw, and the procedures for maintaining confidentiality to the students and their parents. After confirming that each student understood the study information and voluntarily agreed to participate, both the student and a parent signed written informed consent forms. All data were anonymized and used solely for academic research purposes. Findings were reported only in aggregate form, and no information that could identify individual participants was disclosed.

### 2.4. Data Analysis

The analysis included 52 participants. Because match and nonmatch trials were unequal, balanced accuracy, calculated as the mean of the hit and correct-rejection rates, was used as the primary updating measure; overall accuracy was examined in sensitivity analyses. Descriptive statistics and Pearson correlations were also calculated.

A parallel statistical indirect-association model (PROCESS Model 4) examined statistical indirect associations among updating, semantic radical awareness, phonetic radical awareness, and reading comprehension, with semantic and phonetic radical awareness entered as parallel intermediate variables from 5000 bootstrap samples. A conditional statistical indirect-association model (PROCESS Model 14) examined whether communication mode (0 = no daily sign language use, 1 = daily sign language use) moderated the association between semantic radical awareness and reading comprehension. Semantic radical awareness was mean-centered, and group-specific indirect associations and the index of moderated indirect association were estimated. Given the cross-sectional design, all indirect associations were interpreted as statistical associations. The two communication-mode groups were compared on background characteristics, and the moderation model was re-estimated controlling for hearing-loss severity, grade, hearing-assistive device use, and school fixed effects. Because this analysis was exploratory and the sample was limited, no model-specific a priori power analysis was conducted; estimates were interpreted with emphasis on effect sizes and bootstrap confidence intervals. Robustness analyses were conducted in separate steps. HC3 heteroskedasticity-robust standard errors were used for the base model and for a model additionally adjusting for age and sex. Huber M-estimation was used as a separate check of robustness to potentially influential observations. The analyses were also repeated using overall 2-back accuracy instead of balanced accuracy. Finally, the exploratory moderated model was re-estimated in a fully adjusted specification controlling for hearing-loss severity, grade, hearing-assistive device use, age, hearing-loss onset, and school fixed effects.

## 3. Results

### 3.1. Descriptive Statistics and Correlation Analyses

As shown in Table 2, the mean balanced accuracy of the 2-back task was 50.38% (*SD* = 15.24). The mean hit rate was 38.46% (*SD* = 19.59), the mean false-alarm rate was 25.32% (*SD* = 15.66), and the nonmatch omission rate was 12.37% (*SD* = 13.52). The mean correct-rejection rate was 62.31% (*SD* = 18.97), and the mean signal-detection sensitivity was d′ = 0.43 (*SD* = 0.87), the response-conditional sensitivity check is d′ = 0.45 (*SD* = 0.95). Given the overall omission rate of 12.12%, d′ may reflect both discrimination and response completion. We therefore interpret d′ as a descriptive measure of task sensitivity rather than a pure measure of updating ability. Details are provided in Supplementary Table S5.

Balanced accuracy was close to the 50% benchmark (*M* = 0.504, *SD* = 0.152), but the distribution showed substantial individual variability (median = 0.475, IQR = 0.181, range = 0.242–0.892). Thirty-three participants (63.5%) scored below the 50% benchmark, whereas 19 participants (36.5%) scored above it. Thus, the updating measure reflected heterogeneous individual differences in 2-back task sensitivity rather than uniformly above-chance performance.

Balanced accuracy was positively correlated with semantic radical awareness (*r* = 0.387, *p* = 0.005) and phonetic radical awareness (*r* = 0.388, *p* = 0.006). Semantic radical awareness was also positively correlated with reading comprehension (*r* = 0.407, *p* < 0.01). By contrast, neither the correlation between updating and reading comprehension (*r* = 0.153, *p* = 0.279) nor that between phonetic radical awareness and reading comprehension (*r* = 0.156, *p* = 0.271) was significant. The combined radical-awareness score, calculated from the semantic and phonetic radical-awareness subtests, was positively correlated with reading comprehension (*r* = 0.299, *p* < 0.05). Overall accuracy was retained as a secondary updating measure and was examined in sensitivity analyses (Table S4).

To examine the distributional characteristics of the primary measures, we reported minimum and maximum scores, skewness, kurtosis, and the occurrence of extreme scores for semantic radical awareness, phonetic radical awareness, and reading comprehension. Semantic radical awareness scores were relatively evenly distributed, and no participant scored 0. Phonetic radical awareness scores showed little skewness but some clustering at the lower end of the distribution; 10 students (19.2%) scored 0. Raw reading comprehension scores ranged from 0 to 18, whereas the within-grade standardized scores ranged from −1.433 to 3.113, and showed some positive skew (skewness = 1.011, kurtosis = 0.334). Only one student (1.9%) scored 0, and no clear floor effect was evident. Details are presented in Table 3.

### 3.2. Parallel Indirect Associations Between Updating and Reading Comprehension

PROCESS Model 4 was used to estimate a parallel statistical indirect-association model that included semantic and phonetic radical awareness simultaneously, based on 5000 bootstrap samples. The variance inflation factors ranged from 1.22 to 2.00, and the tolerance values ranged from 0.50 to 0.82. These values did not indicate severe multicollinearity among the predictors. Semantic and phonetic radical awareness were strongly correlated (*r* = 0.695). However, collinearity diagnostics indicated that severe multicollinearity was unlikely (Table S5). The negative coefficient for phonetic radical awareness in the parallel model may therefore reflect shared variance or a suppression-like effect rather than a negative association with reading comprehension.

As reported in Table 4, the total association between updating and reading comprehension was not significant (*B* = 0.003, *SE* = 0.009, *p* = 0.730), nor was the direct association (*B* = −0.005, *SE* = 0.010, *p* = 0.617). Updating was significantly and positively associated with semantic radical awareness (*B* = 0.257, *SE* = 0.092, *p* = 0.008) and phonetic radical awareness (*B* = 0.253, *SE* = 0.103, *p* = 0.018). When both radical-awareness measures were included in the same model, semantic radical awareness was significantly and positively associated with reading comprehension (*B* = 0.054, *SE* = 0.018, *p* = 0.004), whereas the association between phonetic radical awareness and reading comprehension was not significant *(B* = −0.023, *SE* = 0.016, *p* = 0.161).

The specific indirect association through semantic radical awareness was significant (0.014, BootSE = 0.007, 95% CI [0.003, 0.031]), whereas the specific indirect association through phonetic radical awareness was not significant (−0.006, BootSE = 0.006, 95% CI [−0.018, 0.003]). The contrast between the two specific indirect associations was significant (0.020, BootSE = 0.012, 95% CI [0.001, 0.047]), whereas the total indirect association was not significant (0.008, BootSE = 0.006, 95% CI [−0.001, 0.021]).

### 3.3. Exploratory Conditional Indirect Associations Across Communication-Mode Groups

An exploratory PROCESS Model 14 analysis examined whether broad communication-mode group, indexed by daily sign language use, moderated the association between semantic radical awareness and within-grade standardized reading comprehension (Table 5). Daily sign language use was coded as 0 = no daily use and 1 = daily use. Semantic radical awareness was mean-centered before the interaction term was constructed. The interaction was significant (*B* = 0.058, *SE* = 0.026, *t* = 2.216, *p* = 0.032, 95% CI [0.005, 0.111]). The simple slope was not significant among students who did not use sign language daily (*B* = 0.006, *SE* = 0.020, *t* = 0.307, *p* = 0.760, 95% CI [−0.034, 0.047]), but was positive and significant among daily sign language users (*B* = 0.064, *SE* = 0.018, *t* = 3.619, *p* < 0.001, 95% CI [0.029, 0.100]).

Table 6 presents the conditional indirect association through semantic radical awareness was not significant among students who did not use sign language daily (effect = 0.002, BootSE = 0.005, 95% CI [−0.007, 0.012]). It was significant among daily sign language users (effect = 0.017, BootSE = 0.007, 95% CI [0.005, 0.034]). The index of moderated indirect association was significant (index = 0.015, BootSE = 0.007, 95% CI [0.004, 0.032]).

**Figure 1:**
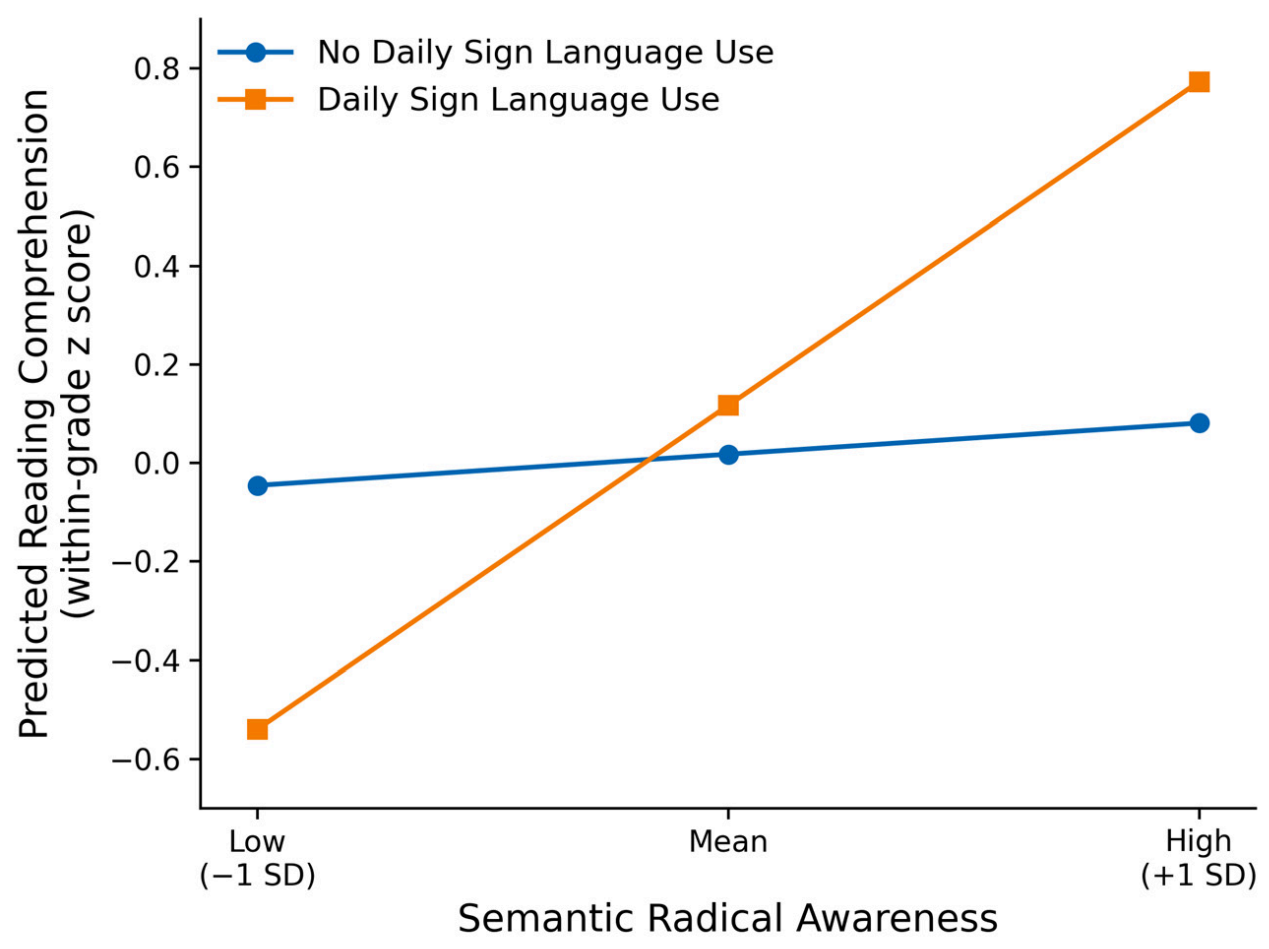
Moderating effect of daily sign language use on the association between semantic radical awareness and reading comprehension. Predicted values are shown at low (−1 *SD*), mean, and high (+1 *SD*) levels of semantic radical awareness, with updating held at its mean.

This result indicates that the statistical indirect association between updating and reading comprehension through semantic radical awareness varied across the communication-mode group (Figure 1). Given the modest sample size (N = 52), including 22 students who did not use sign language in daily life and 30 students who did, the estimates from the moderated indirect-association model should be interpreted as exploratory evidence.

As a robustness analysis, the two daily sign language use groups were compared on background characteristics, and the moderation model was re-estimated with the prespecified covariates and school fixed effects. The groups were comparable in school distribution, hearing-loss severity, grade, age, sex, and hearing-loss onset, but differed in hearing-assistive device use, χ^2^(2) = 12.249, *p* = 0.002 (Table S6). After adjustment, the interaction between semantic radical awareness and daily sign language use remained significant (*B* = 0.053, 95% CI [0.002, 0.104]), as did the index of moderated indirect association (index = 0.015, 95% CI [0.001, 0.029]). The conditional indirect association was significant among daily sign language users but not among nonusers. School effects were nonsignificant in the mediator equation; in the outcome equation, School 2 scored higher than School 1, whereas School 3 did not differ from School 1.

### 3.4. Sensitivity Analyses

To assess the robustness of the main findings, the analyses were repeated using HC3 robust standard errors and Huber M-estimation, with age and gender included as covariates. Across estimation methods and covariate specifications, the positive association between semantic radical awareness and reading comprehension remained significant. The interaction between semantic radical awareness and daily sign language use also remained significant, with effects in the same direction as those in the primary models.

Because the 2-back task contained unequal numbers of match and nonmatch trials, the primary analyses were repeated using overall accuracy instead of balanced accuracy. The two scoring measures were highly correlated (*r* = 0.968, *p* < 0.001). When overall accuracy was used, the specific indirect association via semantic radical awareness and the contrast between the specific indirect associations via semantic and phonetic radical awareness remained significant. The interaction between semantic radical awareness and daily sign language use and the index of moderated indirect association also remained significant.

These results indicate that the main pattern did not depend on the 2-back scoring method and was also observed when reading comprehension was standardized within grade. After additionally adjusting for hearing-loss severity, grade, hearing-assistive device use, age, hearing-loss onset, and school fixed effects, the interaction was statistically significant (*B* = 0.053, *p* = 0.042, 95% CI [0.002, 0.104]), and the index of moderated indirect association remained significant (index = 0.015, 95% CI [0.001, 0.029]). Thus, the main findings were generally robust across alternative scoring procedures and measured background characteristics, although the exploratory moderation pattern should be interpreted cautiously. Detailed results are provided in Tables S1–S6 in the Supplementary Materials.

## 4. Discussion

This study examined the association between visual–semantic processing, updating, and reading comprehension among DHH students and addressed five exploratory research questions concerning updating and daily sign language use. Semantic radical awareness was positively associated with reading comprehension, whereas updating was not directly associated with reading comprehension but was positively associated with both semantic and phonetic radical awareness. The statistical indirect association through semantic radical awareness was significant, whereas the corresponding association through phonetic radical awareness was not; the contrast between the two indirect associations was significant. An exploratory difference was also observed between the broadly defined daily sign language use groups: the association between semantic radical awareness and reading comprehension appeared stronger among daily sign language users, with a similar pattern for the conditional indirect association.

### 4.1. Association Between Semantic Radical Awareness and Reading Comprehension

Semantic radical awareness was positively associated with reading comprehension among DHH students. This association remained significant when updating and phonetic radical awareness were included in the same model, whereas the association between phonetic radical awareness and reading comprehension was not statistically significant. Thus, of the two forms of Chinese orthographic information examined, visual–semantic information use, as indexed by semantic radical awareness, showed a more robust association with reading comprehension.

This result is similar to previous findings on deaf readers, who can maintain effective reading by drawing on orthographic and semantic information despite limited phonological processing (Bélanger et al., 2013; Emmorey et al., 2013). It also provides evidence from deaf populations in support of the Triangle Model, specifically the account that when phonological access is constrained, direct semantics–orthography links may carry greater functional weight in reading.

Notably, in this study, phonetic radical awareness scores were substantially lower than semantic radical awareness scores and were clustered toward the lower end of the scale. This pattern may have more than one explanation. Phonetic-radical processing depends more directly on access to phonological representations and pronunciation information, which may be limited or uneven among DHH students. Using a phonetic radical may therefore have been more difficult for some participants. However, the task itself may also have contributed to the result. Where the semantic task relied on recognition, the phonetic task asked participants to produce a pronunciation themselves, either by supplying pinyin or by offering a familiar homophone. The lower scores and restricted range may have reduced the ability to detect an association with reading comprehension. Thus, the nonsignificant association involving phonetic radical awareness should not be taken as evidence that phonetic radical awareness is unrelated to reading comprehension. Because the study did not include a hearing comparison group and the two tasks differed in format and demands, the observed contrast cannot be attributed entirely to differences in semantic and phonological processing. Even with these qualifications, semantic radical awareness remained significantly and positively associated with reading comprehension, whereas the association involving phonetic radical awareness was not significant.

### 4.2. Indirect Association Between Updating and Reading Comprehension via Semantic Radical Awareness

Parallel indirect-association analysis showed no significant direct association between updating and reading comprehension. However, the specific indirect association via semantic radical awareness was significant, whereas the indirect association via phonetic radical awareness was not. The difference between the two indirect associations was significant.

Although the sample performed only modestly overall on the updating task, individual differences in updating did track radical awareness: students with more efficient updating showed comparatively stronger awareness of character components. Such a pattern is consistent with the view that updating resources support access to character-level information, particularly the visual–semantic cues that underpin reading comprehension. The conclusion warrants caution, however—performance on the updating task may draw on processes other than updating per se.

These findings place semantic radical awareness at the center of the observed association pattern. Updating was positively associated with both semantic and phonetic radical awareness, but only semantic radical awareness was independently associated with reading comprehension and showed a significant specific indirect association involving updating. The contrast between the semantic and phonetic indirect associations was significant, although the combined indirect association was not. Thus, under the present measurement conditions, visual–semantic processing, indexed by semantic radical awareness, showed a distinctive statistical relevance to reading comprehension in this DHH sample.

For DHH readers, variation in language experience may affect the accessibility of the lexical, syntactic, and semantic representations required for comprehension (Volpato, 2019). When access to word meanings is less efficient, relations across sentences are harder to identify, or lexical, syntactic, and semantic information is not fully integrated, readers may have difficulty constructing a coherent representation of the text (Olujić Tomazin et al., 2025; Sehyr & Emmorey, 2022). Differences in lexical-semantic access and sentence-level integration may therefore have limited the extent to which updating resources were reflected directly in reading comprehension. This offers one explanation for why, in the present DHH sample, updating related to semantic and phonetic radical awareness yet showed no direct association with reading comprehension.

At the same time, the present results suggest a more specific language-specific route through which updating may still be related to reading comprehension: the coordination of domain-general updating with visual–semantic processing. Updating requires readers to maintain newly encountered information, replace no-longer-relevant representations, and coordinate current information with previously processed content. Semantic radical awareness reflects sensitivity to visual cues that constrain the meanings of Chinese characters (Tong et al., 2021; Y. Li et al., 2022). When updating, resources engaged in this visual–semantic mapping may help readers maintain candidate meanings, revise these meanings as contextual information accumulates, and connect word-level meanings across sentences (Zhang et al., 2024). These operations may offer a plausible route through which updating can be statistically linked to the construction of a coherent text representation and, consequently, to reading comprehension. Phonetic radical awareness may occupy a less direct position in this process because phonetic radicals tend to provide pronunciation-related cues, whereas comprehension may depend more directly on the activation and integration of lexical-semantic information. Thus, visual–semantic processing may function as a language-specific interface through which domain-general updating becomes relevant to reading comprehension in DHH students. This account provides a theoretically plausible interpretation of the observed statistical pattern, although the cross-sectional design does not establish the proposed mechanism as causal.

In the present study, this divergence centered on the semantic information carried by semantic radicals. The association between updating and semantic radical awareness suggests that domain-general executive resources may be involved in visual–semantic processing. The independent association between semantic radical awareness and reading comprehension further suggests that these resources may be linked to reading comprehension through this specific form of visual–semantic processing. By contrast, updating was also associated with phonetic radical awareness, but phonetic radical awareness was not associated with reading comprehension. This pattern is consistent with the functional distinction discussed above. Among DHH students, it may not be overall performance across the two radical-awareness measures, but rather the visual–semantic information indexed by semantic radical awareness that is associated with reading comprehension, but rather the visual–semantic information within the orthographic system that supports access to meaning. In this sense, the findings are consistent with a possible resource translation account. Resource translation does not imply that domain-general executive resources are converted directly into reading ability. Rather, it refers to the possibility that domain-general updating ability may be indirectly associated with reading comprehension via a language-specific process such as semantic radical awareness. This pattern resembles the structure reported by Zhao and Wu (2021) for the association between visual processing and reading performance among Chinese deaf children.

Because the study was cross-sectional, the observed indirect associations do not establish temporal or causal relationships. The pathway contrast should also be interpreted cautiously, for three reasons: the two tasks differed in format; the phonetic task yielded relatively low scores, although this does not conclusively establish a floor effect; and the sign reversal of the phonetic coefficient may reflect suppression-like or competitive effects arising from shared variance. Thus, the contrast represents a conditional statistical comparison under the present measurement conditions, and the proposed role of lexical-semantic access and sentence-level integration remains tentative because these processes were not directly measured.

### 4.3. Communication Mode as an Exploratory Contextual Factor

An exploratory difference was observed between the broadly defined communication-mode groups in the association between semantic radical awareness and reading comprehension. This association appeared stronger among students who used sign language in daily life, and the corresponding conditional indirect association showed a similar pattern. This pattern suggests that the association between visual–semantic information and reading comprehension may vary across students with different everyday language-use backgrounds.

From the perspective of the Triangle Model, the relative contributions of orthographic, phonological, and semantic information to reading may vary according to information availability and language experience (Harm & Seidenberg, 2004; Seidenberg & McClelland, 1989). For DHH students, access to auditory-phonological information may be constrained to some extent, whereas sign language involves deriving lexical and semantic information from visually accessible linguistic forms. Although sign language and semantic radicals in Chinese characters differ substantially in their linguistic structure, both involve mappings between visual forms and linguistic meaning. Students who use sign language in daily life may therefore have more experience with visual form-to-meaning mappings, which could be related to the role of semantic radical cues during Chinese reading. This interpretation remains tentative and should be examined in larger samples using more detailed measures of language experience.

### 4.4. Research Significance and Educational Implications

The findings indicate that semantic radical awareness was not only positively associated with reading comprehension among DHH students but may also represent a specific language-processing link in the indirect association between updating and reading comprehension. The exploratory difference between the daily sign language use groups further suggests that this association may vary according to students’ patterns of daily language use. Taken together, these findings provide correlational evidence relevant to understanding individual differences in reading comprehension among DHH students from the perspective of visual–semantic information use.

In educational practice, educators might consider explicitly teaching the semantic functions of radicals as part of Chinese character recognition and reading instruction. Such instruction may help students connect visual forms with character meanings and integrate semantic radical cues into word and text comprehension. Teachers should also attend to students’ language-use patterns and consider these differences when providing instructional support. However, daily sign language use itself should not be regarded as a direct means of improving reading comprehension. Because the present study did not directly evaluate an instructional intervention, these educational implications should be considered preliminary and require further examination in intervention studies.

### 4.5. Study Limitations and Future Directions

Several limitations should be noted. First, the convenience sample was small (*N* = 52; group sizes = 22 and 30), limiting the precision and stability of the exploratory conditional indirect-association estimates. The attenuation of the interaction after covariate adjustment further supports cautious interpretation and the need for replication in larger, more balanced samples. Second, the cross-sectional, single-session design limits conclusions about temporal ordering and causal processes. Third, although the researcher-developed measures showed acceptable reliability and expert review, their validity and the equivalence of the grade-specific reading tests require further evaluation. The semantic and phonetic tasks also differed in format, and phonetic scores were relatively low, which may have influenced the pathway contrast, though this does not conclusively establish a floor effect. Fourth, because no hearing comparison group was included, the study cannot determine whether DHH students show stronger visual–semantic processing than hearing peers. Fifth, split-half reliability does not establish construct validity. Future studies should include multiple updating tasks together with independent measures of attention, processing speed, and working-memory updating to examine convergent and discriminant validity in larger samples. Finally, daily sign language use was defined broadly and did not capture age of exposure, frequency, proficiency, or language quality; unmeasured differences in language experience and cognitive development may therefore remain. Future research should use larger samples, longitudinal designs, validated measures, and more detailed assessments of language experience.

## 5. Conclusions

This study focused on visual–semantic processing in reading among DHH students. The findings suggest that semantic radical awareness may be a particularly relevant correlate of reading comprehension and may represent a language-specific link in the exploratory statistical indirect association between updating and reading comprehension. This pattern highlights the potential value of visual–semantic processing for understanding reading comprehension among DHH students. The exploratory difference between the broadly defined daily sign-language-use groups further suggests that this association may vary with students’ language-use backgrounds, without establishing a specific effect of sign-language experience. Given the small cross-sectional sample, these findings should be interpreted as preliminary statistical associations and examined further in larger longitudinal studies.

## Figures and Tables

**Table 1:** Participant characteristics (N = 52).

Characteristic	Category or Statistic	*n* (%) or *M (SD)*
Age (years)	Mean ± *SD*	16.08 (1.61)
	Range	13–19
Sex	Male	27 (51.9%)
	Female	25 (48.1%)
Hearing loss severity	Mild	7 (13.5%)
	Moderate	16 (30.8%)
	Severe	10 (19.2%)
	Profound	19 (36.5%)
Daily sign language use	No daily sign language use	22 (42.3%)
	Daily sign language use	30 (57.7%)
Grade	Grade 7	7 (13.5%)
	Grade 8	11 (21.2%)
	Grade 9	34 (65.4%)

Daily sign language use was classified according to participants’ current primary communication mode. Participants who primarily used spoken language without sign language were classified as having no daily sign language use, whereas those who primarily used sign language or used both sign and spoken language were classified as having daily sign language use.

**Table 2:** Descriptive statistics and correlations among study variables (*N* = 52). (**A**) Descriptive statistics for 2-back performance indicators. (**B**) Distribution of 2-back balanced accuracy and performance relative to the 50% benchmark (N = 52). (**C**) Descriptive statistics and correlations among the primary study variables.

(**A**)							
**Measure**	***M***	***SD***	**Min**	**Max**	**Unit/Denominator**		
Balanced accuracy	50.38	15.24	24.17	89.17	%		
Overall accuracy	54.36	15.62	26.67	91.11	%		
Hit rate	38.46	19.59	6.67	86.67	% of 30 match trials		
False-alarm rate	25.32	15.66	0.00	53.33	% of 60 nonmatch trials		
Correct-rejection rate	62.31	18.97	23.33	96.67	% of 60 nonmatch trials		
Nonmatch omission rate	12.37	13.52	0.00	73.33	% of 60 nonmatch trials		
Match omission rate	11.60	11.93	0.00	53.33	% of 30 match trials		
Overall omission rate	12.12	12.73	0.00	66.67	% of 90 trials		
d′, fixed planned-trial denominators	0.43	0.87	−0.88	3.33	log-linear correction		
d′, response-conditional sensitivity check	0.45	0.95	−0.97	3.46	omissions excluded		
(**B**)							
**Measure**					**Results**		
Mean balanced accuracy					0.504 (50.38%)		
Participants at or below the 50% benchmark, n (%)					33 (63.5%)		
Participants below the 50% benchmark, n (%)					33 (63.5%)		
Participants above the 50% benchmark, n (%)					19 (36.5%)		
Mean balanced accuracy among participants below the 50% benchmark					0.411 (41.06%)		
Mean balanced accuracy among participants above the 50% benchmark					0.666 (66.58%)		
Median balanced accuracy					0.475 (47.50%)		
Interquartile range (IQR)					0.181 (18.13 percentage points)		
Q1–Q3					0.398–0.579		
Range					0.242–0.892 (24.17–89.17%)		
(**C**)							
**Variable**	***M***	***SD***	**1**	**2**	**3**	**4**	**5**
1. Updating (2-back balanced accuracy, %)	50.38	15.24	1				
2. Semantic radical awareness	28.81	10.23	0.387 **	1			
3. Phonetic radical awareness	12.79	11.28	0.388 **	0.695 **	1		
4. Semantic + phonetic radical-awareness score	41.60	19.81	0.421 **	0.912 **	0.929 **	1	
5. Reading comprehension	0.00	0.98	0.153	0.407 **	0.156	0.299 *	1

Note. Match and nonmatch trials used different denominators. Hit rate was calculated as n(F response on match trials)/30; match miss rate as [n(J response on match trials) + n(match omissions)]/30; false-alarm rate as n(F response on nonmatch trials)/60; correct-rejection rate as n(J response on nonmatch trials)/60; match omission rate as n(match omissions)/30; and nonmatch omission rate as n(nonmatch omissions)/60. Thus, false-alarm rate + correct-rejection rate + nonmatch omission rate = 100% (up to rounding), whereas false-alarm rate + correct-rejection rate alone does not. Balanced accuracy was (hit rate + correct-rejection rate)/2. Overall accuracy was [n(F response on match trials) + n(J response on nonmatch trials)]/90, with omissions scored as errors. Signal-detection sensitivity was d′ = Φ^−1^(H*) − Φ^−1^(FA*), using the log-linear continuity correction H* = (hits + 0.5)/(30 + 1) and FA* = (false alarms + 0.5)/(60 + 1). Note. Balanced accuracy was calculated as the mean of the hit rate and correct-rejection rate, with omissions counted as errors. Classification relative to the 50% benchmark was based on unrounded participant-level scores. No participant scored exactly 50%. Q1 and Q3 denote the first and third quartiles, respectively. Note. *M* = mean; *SD* = standard deviation. Balanced accuracy was calculated as the mean of the hit rate for match trials and the correct-rejection rate for nonmatch trials. Hit rate was calculated as the proportion of match trials on which participants pressed “F,” and false-alarm rate as the proportion of nonmatch trials on which participants pressed “F.” Omissions were treated as errors in accuracy-based measures. Signal-detection sensitivity was calculated as d′ = z (hit rate) − z (false-alarm rate), with a continuity correction applied when a rate was 0 or 1. Overall accuracy was retained as a secondary measure for sensitivity analyses.* *p* ≤ 0.05 (two-tailed); ** *p* < 0.01 (two-tailed).

**Table 3:** Distributional characteristics of semantic radical awareness, phonetic radical awareness, and within-grade standardized reading comprehension scores.

Measure	N	Min	Max	Skewness	Kurtosis	Score of 0, n (%)	Near-Ceiling Score, n (%)
Semantic Radical Awareness	52	4	51	−0.174	−0.302	0 (0.0%)	0 (0.0%)
Phonetic Radical Awareness	52	0	34	0.343	−1.279	10 (19.2%)	0 (0.0%)
Reading Comprehension	52	−1.433	3.113	1.154	0.971	1 (1.9%)	0 (0.0%)

Note. Reading comprehension is expressed as a within-grade z-score. The raw score of 0 and raw near-ceiling indicators are retained for reference. The theoretical score ranges were 0–60 for semantic and phonetic radical awareness and 0–24 for the raw reading comprehension test. Near-ceiling scores were defined as ≥90% of the theoretical maximum.

**Table 4:** Parallel statistical indirect-association model of semantic and phonetic radical awareness.

Path or Association	*B*/Effect	*SE*/BootSE	*t*	*p*	95% CI
Balanced Accuracy → Semantic Radical Awareness (a_1_)	0.257	0.092	2.788	0.008	[0.072, 0.442]
Balanced Accuracy → Phonetic Radical Awareness (a_2_)	0.253	0.103	2.446	0.018	[0.045, 0.460]
Semantic Radical Awareness → Reading Comprehension (b_1_)	0.054	0.018	3.007	0.004	[0.018, 0.090]
Phonetic Radical Awareness → Reading Comprehension (b_2_)	−0.023	0.016	−1.423	0.161	[−0.055, 0.009]
Balanced Accuracy → Reading Comprehension (Direct Association, c′)	−0.005	0.010	−0.504	0.617	[−0.024, 0.014]
Balanced Accuracy → Reading Comprehension (Total Association, c)	0.003	0.009	0.347	0.730	[−0.016, 0.022]
Specific Indirect Association via Semantic Radical Awareness (a_1_b_1_)	0.014	0.007	—	—	[0.003, 0.031]
Specific Indirect Association via Phonetic Radical Awareness (a_2_b_2_)	−0.006	0.006	—	—	[−0.018, 0.003]
Contrast of Specific Indirect Associations: Semantic—Phonetic	0.020	0.012	—	—	[0.001, 0.047]
Total Indirect Association	0.008	0.006	—	—	[−0.001, 0.021]

Note. Balanced accuracy was calculated as the mean of the hit rate for match trials and the correct-rejection rate for nonmatch trials. Reading comprehension was expressed as a within-grade standardized score. Standard errors and confidence intervals for regression coefficients are based on ordinary least-squares estimation. Indirect associations and their 95% confidence intervals were estimated using 5000 bootstrap resamples. BootSE = bootstrap standard error; CI = confidence interval. No covariates were included in this primary parallel indirect-effect model. Covariate-adjusted analyses were conducted separately as robustness analyses.

**Table 5:** Exploratory difference between communication-mode groups in the association between semantic radical awareness and reading comprehension.

Effect	*B*/Effect	*SE*/BootSE	*t*	*p*	95% CI
Balanced Accuracy	−0.009	0.009	−0.919	0.363	[−0.027, 0.010]
Semantic Radical Awareness	0.006	0.020	0.307	0.760	[−0.034, 0.047]
Daily Sign Language Use	0.099	0.263	0.376	0.709	[−0.430, 0.628]
Semantic Radical Awareness × Daily Sign Language Use	0.058	0.026	2.216	0.032	[0.005, 0.111]
Conditional Indirect Association: No Daily Sign Use	0.002	0.005	—	—	[−0.007, 0.012]
Conditional Indirect Association: Daily Sign Use	0.017	0.007	—	—	[0.005, 0.034]
Index of Moderated Indirect Association	0.015	0.007	—	—	[0.004, 0.032]
Simple Slope: No Daily Sign Language Use	0.006	0.020	0.307	0.760	[−0.034, 0.047]
Simple Slope: Daily Sign Language Use	0.064	0.018	3.619	<0.001	[0.029, 0.100]

Note. Balanced accuracy was used as the primary index of updating. Semantic radical awareness was mean-centered before constructing the interaction term. Daily sign language use was coded as 0 = no daily sign language use and 1 = daily sign language use. Reading comprehension was expressed as a within-grade standardized score.

**Table 6:** Conditional indirect associations and index of moderated indirect association by daily sign language use.

Daily Sign Language Use/Effect	Conditional Indirect Association	BootSE	BootLLCI	BootULCI
No Daily Sign Language Use	0.002	0.005	−0.007	0.012
Daily Sign Language Use	0.017	0.007	0.005	0.034
Index of Moderated Indirect Association	0.015	0.007	0.004	0.032

Note. Balanced accuracy was used as the primary index of updating. Daily sign language use was coded as 0 = no daily sign language use and 1 = daily sign language use. Conditional indirect associations and the index of moderated indirect association were estimated using 5000 bootstrap resamples. BootSE = bootstrap standard error; BootLLCI = lower limit of the 95% bootstrap confidence interval; BootULCI = upper limit of the 95% bootstrap confidence interval.

## Data Availability

The raw data supporting the conclusions of this article will be made available by the authors on request.

## Supplementary Materials

The following supporting information can be downloaded at: https://www.mdpi.com/article/10.3390/bs16101910/s1. Table S1. Sensitivity analyses of the semantic-radical indirect association and the moderating effect of daily sign language use. Table S2. Descriptive statistics for reading comprehension by grade. Table S3. Moderated indirect-association analyses using within-grade standardized reading comprehension scores. Table S4. Sensitivity analyses using overall 2-back accuracy. Panel A. Trial-level scoring rules and denominators. Panel B. Pooled response counts. Panel C. Parallel indirect-effect model. Panel D. Moderated mediation model. Table S5. Collinearity and suppression diagnostics for the parallel statistical indirect-association model. Panel A. Predictor correlations and collinearity statistics. Panel B. Comparison of phonetic radical awareness coefficients. Table S6. Background characteristics and covariate-adjusted exploratory moderated indirect-association analysis. Panel A. Background characteristics by daily sign language use. Panel B. Covariate-adjusted mediator equation predicting semantic radical awareness. Panel C. Covariate-adjusted outcome equation predicting within-grade standardized reading comprehension. Panel D. Conditional indirect associations and index of moderated indirect association.
