# Supplementary material for: Visual–Semantic Processing and Reading Comprehension in Chinese Deaf and Hard-of-Hearing Students: Roles of Updating, Semantic Radical Awareness, and Daily Sign Language Use
Source: Behav Sci (Basel). 2026 Oct 15;16(10):1910. doi: 10.3390/bs16101910 (PMC13701981; doi:10.3390/bs16101910)
Supplement: Supplementary file 1 [file behavsci-16-01910-s001.zip › behavsci-4585441-supplementary.pdf]

Supplementary Materials

Table S1. Sensitivity analyses of the semantic-radical indirect association and the moderating effect of daily sign language use.

Sensitivity Analysis	Semantic Radical Awareness → Reading	Semantic Radical Awareness × Daily Sign
Base model (HC3 robust SEs)	0.054 (0.020), [0.015, 0.093], .008	0.058 (0.022), [0.014, 0.102], .010
Adjusted for age and sex	0.054 (0.021), [0.012, 0.096], .014	0.065 (0.024), [0.016, 0.115], .011
Huber M-estimator	0.052 (0.022), [0.009, 0.094], .018	0.052 (0.020), [0.013, 0.091], .009

Note. Reading comprehension was expressed as a within-grade standardized score. The first effect column reports the association between semantic radical awareness and reading comprehension, controlling for updating and phonetic radical awareness. The second effect column reports the interaction between semantic radical awareness and daily sign language use, controlling for updating and the corresponding lower-order terms. The base and age- and sex-adjusted models were estimated using HC3 heteroskedasticity-robust standard errors. The Huber M-estimator was additionally used to assess robustness to potentially influential observations. Daily sign language use was coded as 0 = no daily sign language use and 1 = daily sign language use.

Table S2. Descriptive Statistics for Reading Comprehension by Grade.

Grade	*n*	*M*	*SD*	*Min*	*Max*
Grade 7	7	4.57	3.10	1	10
Grade 8	11	4.73	3.44	1	12
Grade 9	34	6.21	4.25	0	18

Note. Raw reading comprehension scores are presented. Scores were standardized within grade and used as the primary outcome in the main regression and indirect-association analyses.

Table S3. Moderated indirect-association analyses using within-grade standardized reading comprehension scores.

Effect	*B* / Effect	*SE* / BootSE	*t*	*p*	95% CI
Semantic Radical Awareness × Daily Sign Language Use	0.058	0.026	2.216	.032	[0.005, 0.111]
Simple Slope: No Daily Sign Language Use	0.006	0.020	0.307	.760	[−0.034, 0.047]
Simple Slope: Daily Sign Language Use	0.064	0.018	3.619	< .001	[0.029, 0.100]
Conditional Indirect Effect: No Daily Sign Language Use	0.002	0.005	—	—	[−0.007, 0.012]
Conditional Indirect Effect: Daily Sign Language Use	0.017	0.007	—	—	[0.005, 0.034]
Index of Moderated Mediation	0.015	0.007	—	—	[0.004, 0.032]

Note. Daily sign language use was coded as 0 = no daily sign language use and 1 = daily sign language use. Reading comprehension scores were standardized within grade. Conditional indirect effects and the index of moderated mediation were estimated using 5,000 bootstrap samples.

Table S4. Sensitivity analyses using overall 2-back accuracy.

Panel A. Trial-level scoring rules and denominators

Index	Formula	Denominator	Treatment of omissions	Meaning
Hit rate (H)	n(F response ∣ match) / 30	30 match trials	Omissions are not hits and remain in the denominator.	Proportion of match trials correctly endorsed.
Match miss rate	[n(J response ∣ match) + n(omission ∣ match)] / 30	30 match trials	Match omissions are errors and are included in the miss rate.	Complement of H.
False-alarm rate (FA)	n(F response ∣ nonmatch) / 60	60 nonmatch trials	Nonmatch omissions are separate from FA and remain in the denominator.	Proportion of nonmatch trials incorrectly endorsed.
Correct-rejection rate (CR)	n(J response ∣ nonmatch) / 60	60 nonmatch trials	Nonmatch omissions are not CRs.	Proportion of nonmatch trials correctly rejected.
Match omission rate	n(omission ∣ match) / 30	30 match trials	Reported separately from wrong-key misses.	No-response frequency on match trials.
Nonmatch omission rate	n(omission ∣ nonmatch) / 60	60 nonmatch trials	Reported separately from FA and CR.	No-response frequency on nonmatch trials.
Balanced accuracy	(H + CR) / 2	Condition-balanced	Both omission types lower the relevant condition-specific rate.	Primary updating index.
Overall accuracy	[n(F ∣ match) + n(J ∣ nonmatch)] / 90	90 scorable trials	All omissions are scored as errors.	Secondary index.
Signal-detection sensitivity (d′)	$\Phi^{-1}(H^*) - \Phi^{-1}(FA^*)$	H* and FA*	Omissions are not recoded as hits, FAs, or CRs; they remain represented through the fixed planned-trial	Descriptive sensitivity; not a pure updating measure when

denominators.	omissions are substantial.

Panel B. Pooled response counts

Condition	F response	J response	Omission	Total	Percentages within condition
Match (30 per participant)	600 hits	779 wrong-key responses	181 match omissions	1,560	38.46% + 49.94% + 11.60% = 100%
Nonmatch (60 per participant)	790 false alarms	1,944 correct rejections	386 nonmatch omissions	3,120	25.32% + 62.31% + 12.37% = 100%

Panel C. Parallel indirect-effect model.

Path or Effect	*B* / Effect	*SE* / BootSE	*t*	*p*	95% CI
Updating → Semantic Radical Awareness (a_1)	0.258	0.089	2.903	.005	[0.079, 0.436]
Updating → Phonetic Radical Awareness (a_2)	0.248	0.100	2.485	.016	[0.048, 0.449]
Semantic Radical Awareness → Reading Comprehension (b_1)	0.056	0.018	3.164	.003	[0.021, 0.092]
Phonetic Radical Awareness → Reading Comprehension (b_2)	−0.022	0.016	−1.382	.173	[−0.054, 0.010]
Updating → Reading Comprehension (Direct Association, c′)	−0.010	0.009	−1.099	.277	[−0.029, 0.008]
Updating → Within-Grade Standardized Reading Comprehension (Total Association, c)	−0.001	0.009	−0.111	.912	[−0.020, 0.017]
Total Indirect Effect	0.009	0.006	—	—	[−0.001, 0.022]
Specific Indirect Association via Semantic Radical Awareness (a_1b_1)	0.014	0.007	—	—	[0.003, 0.032]
Specific Indirect Association via Phonetic Radical Awareness (a_2b_2)	−0.005	0.005	—	—	[−0.018, 0.003]
Contrast of Specific Indirect Association: Semantic – Phonetic	0.020	0.011	—	—	[0.002, 0.046]

Panel D. Moderated mediation model

Predictor / Effect	*B*	*SE*	*t*	*p*	95% CI
Updating	−0.014	0.009	−1.507	.139	[−0.032, 0.005]
Semantic Radical Awareness	0.008	0.020	0.407	.686	[−0.032, 0.048]
Daily Sign Language Use	0.081	0.260	0.313	.756	[−0.441, 0.604]
Semantic Radical Awareness × Daily Sign Language Use	0.060	0.026	2.314	.025	[0.008, 0.112]
Simple Slope: No Daily Sign Language Use	0.008	0.020	0.407	.686	[−0.032, 0.048]
Simple Slope: Daily Sign Language Use	0.068	0.018	3.870	< .001	[0.033, 0.103]
Conditional indirect association: No daily sign language use	0.002	0.005	—	—	[−0.007, 0.012]
Conditional indirect association: Daily sign language use	0.018	0.008	—	—	[0.006, 0.035]
Index of moderated indirect association	0.015	0.008	—	—	[0.004, 0.032]

Note. Semantic radical awareness was mean-centered before constructing the interaction term. Daily sign language use was coded as 0 = no daily sign language use and 1 = daily sign language use. Unstandardized coefficients are reported.

Table S5. Collinearity and suppression diagnostics for the parallel statistical indirect-association model.

Panel A. Predictor correlations and collinearity statistics.

Predictor	Balanced Accuracy	Semantic Radical Awareness	Phonetic Radical Awareness	VIF	Tolerance
Balanced Accuracy	1.000	0.387	0.388	1.22	0.823
Semantic Radical Awareness	0.387	1.000	0.695	2.00	0.501
Phonetic Radical Awareness	0.388	0.695	1.000	2.00	0.500

Panel B. Comparison of phonetic radical awareness coefficients.

Analysis	Coefficient	*SE*	*t*	*p*	95% CI
Zero-order association with reading comprehension (r)	0.106	—	0.752	.456	—
Single-mediator model, controlling for balanced accuracy	0.009	0.013	0.668	.507	[−0.018, 0.035]
Parallel indirect-effect model,	−0.023	0.016	−1.423	.161	[−0.055, 0.009]

controlling for balanced accuracy and semantic radical awareness	

Note. VIF = variance inflation factor. Tolerance = 1/VIF. Balanced accuracy was used as the primary updating measure. VIFs and tolerance values were calculated for the predictors in the outcome equation containing balanced accuracy, semantic radical awareness, and phonetic radical awareness. The coefficient for phonetic radical awareness changed from positive in the zero-order and single-mediator models to negative in the parallel model. This sign change may reflect shared variance or a suppression-like effect, but it does not establish a statistically significant suppression effect. All regression coefficients are unstandardized.

Table S6. Background characteristics and covariate-adjusted exploratory moderated indirect-association analysis.

Panel A. Background characteristics by daily sign language use.

Characteristic	No Daily Sign Language Use (n = 22)	Daily Sign Language Use (n = 30)	Test Statistic	*p*
School 1 / 2 / 3, n (%)	13 (59.1) / 7 (31.8) / 2 (9.1)	13 (43.3) / 11 (36.7) / 6 (20.0)	$\chi^2(2) = 1.698$	.428
Sex, code 1 / 2, n (%)	11 (50.0) / 11 (50.0)	16 (53.3) / 14 (46.7)	$\chi^2(1) = 0.056$	.812
Hearing-loss severity, levels 1–4, n (%)	4 / 6 / 4 / 8	3 / 10 / 6 / 11	$\chi^2(3) = 0.805$	.848
Grade, levels 1–3, n (%)	2 / 5 / 15	5 / 6 / 19	$\chi^2(2) = 0.631$	.729
Age, *M* (*SD*)	15.91 (1.82)	16.20 (1.45)	Welch's t = −0.619	.540
Hearing-loss onset, levels 1 / 2, n (%)	15 (68.2) / 7 (31.8)	24 (80.0) / 6 (20.0)	$\chi^2(1) = 0.945$	.331
Hearing-assistive device use, levels 1–3, n (%)	12 / 9 / 1	12 / 4 / 14	$\chi^2(2) = 12.249$	.002

Note. The two groups were comparable in school composition, hearing-loss severity, grade, age, sex, and hearing-loss onset, but differed in hearing-assistive device use.

Panel B. Covariate-adjusted mediator equation predicting semantic radical awareness.

Predictor	*B*	*SE*	*t*	*p*	95% CI
Balanced accuracy	0.284	0.091	3.127	.003	[0.101, 0.468]
Hearing-loss severity	−1.894	1.342	−1.411	.166	[−4.602, 0.814]
Grade	6.398	2.001	3.198	.003	[2.361, 10.436]
Assistive device, level 2	5.556	3.365	1.651	.106	[−1.235, 12.348]
Assistive device, level 3	−1.526	3.519	−0.434	.667	[−8.627, 5.574]
School 2	−1.533	2.899	−0.529	.600	[−7.383, 4.318]
School 3	−0.622	3.610	−0.172	.864	[−7.908, 6.664]
Age	−1.187	0.976	−1.216	.231	[−3.155, 0.782]

Predictor	*B*	*SE*	*t*	*p*	95% CI
Hearing-loss onset	5.856	2.985	1.962	.056	[−0.167, 11.879]

Panel C. Covariate-adjusted outcome equation predicting within-grade standardized reading comprehension.

Predictor	*B*	*SE*	*t*	*p*	95% CI
Balanced accuracy	−0.013	0.010	−1.365	.180	[−0.033, 0.006]
Semantic radical awareness (mean-centered)	0.030	0.021	1.398	.170	[−0.013, 0.072]
Daily sign language use	−0.206	0.272	−0.759	.453	[−0.755, 0.343]
Semantic radical awareness × Daily Sign Language Use	0.053	0.025	2.109	.042	[0.002, 0.104]
Hearing-loss severity	0.221	0.132	1.671	.103	[−0.047, 0.489]
Grade	−0.115	0.215	−0.532	.598	[−0.550, 0.321]
Assistive device, level 2	−0.655	0.340	−1.925	.062	[−1.344, 0.033]
Assistive device, level 3	0.233	0.354	0.658	.514	[−0.483, 0.949]
School 2	0.805	0.281	2.869	.007	[0.237, 1.372]
School 3	0.313	0.363	0.862	.394	[−0.421, 1.046]
Age	−0.078	0.098	−0.794	.432	[−0.276, 0.120]
Hearing-loss onset	−0.479	0.300	−1.597	.118	[−1.086, 0.128]

Panel D. Conditional indirect associations and index of moderated indirect association.

Daily Sign Language Use	Indirect Association	BootSE	BootLLCI	BootULCI
No daily sign language use	0.008	0.006	−0.002	0.023
Daily sign language use	0.023	0.008	0.008	0.040
Index of moderated indirect association	0.015	0.007	0.001	0.029

Note. Balanced accuracy was used as the primary updating measure. Reading comprehension was standardized within grade. The fully adjusted model simultaneously controlled for hearing-loss severity, grade, hearing-assistive device use, age, hearing-loss onset, and school fixed effects. Hearing-assistive device use was dummy-coded with level 1 as the reference category, and school was dummy-coded with School 1 as the reference category. Hearing-loss severity and grade were entered as numeric covariates. Semantic radical awareness was mean-centered before constructing the interaction term. Daily sign language use was coded as 0 = no daily sign language use and 1 = daily sign language use. Conditional indirect associations and the index of moderated indirect association were estimated using 5,000 bootstrap resamples. All regression coefficients are unstandardized.
